# Preventing Tumour Recurrence after Liver Transplantation: The Role of Machine Perfusion

**DOI:** 10.3390/ijms21165791

**Published:** 2020-08-12

**Authors:** Yuri Boteon, Mauricio Alfredo Flores Carvalho, Rebecca Panconesi, Paolo Muiesan, Andrea Schlegel

**Affiliations:** 1 Liver Unit, Albert Einstein Hospital, 05652–900 São Paulo, Brazil; yurimed43@yahoo.com.br; 2 Albert Einstein Jewish Institute for Education and Research, 05652–900 São Paulo, Brazil; 3 Hepatobiliary Unit, Department of Clinical and Experimental Medicine, University of Florence, AOU Careggi, 50134 Florence, Italy; drmauras@gmail.com (M.A.F.C.); rebecca.panconesi@stud.unifi.it (R.P.); paolo.muiesan@unifi.it (P.M.); 4 The Liver Unit, Queen Elizabeth Hospital Birmingham, Edgbaston, Birmingham B15 2TH, UK; 5 NIHR Birmingham Biomedical Research Centre, University Hospitals Birmingham NHS Foundation Trust and University of Birmingham, Birmingham B15 2TT, UK

**Keywords:** ischemia reperfusion injury, mitochondria, hepatocellular carcinoma, cancer recurrence, machine perfusion

## Abstract

Tumour recurrence is currently a hot topic in liver transplantation. The basic mechanisms are increasingly discussed, and, for example, recurrence of hepatocellular carcinoma is often described in pre-injured donor livers, which frequently suffer from significant ischemia/reperfusion injury. This review article highlights the underlying mechanisms and describes the specific tissue milieu required to promote tumour recurrence after liver transplantation. We summarise the current literature in this field and show risk factors that contribute to a pro-tumour-recurrent environment. Finally, the potential role of new machine perfusion technology is discussed, including the most recent data, which demonstrate a protective effect of hypothermic oxygenated perfusion before liver transplantation.

## 1. Introduction

Liver transplantation (LT) is the gold standard treatment for end-stage liver diseases and early stage nonresectable hepatocellular carcinoma (HCC). It is a valuable tool for HCC treatment because it removes not only the malignant tumour but also the diseased liver parenchyma, which has the potential to develop new malignant lesions. The number of HCC cases listed for LT are continuously increasing worldwide, being already the leading indication for LT in the United States with approximately 24% of registrations in 2015 [1].

Despite its success and the use of well-established patient selection criteria, based on morphologic tumour characteristics, the reported HCC recurrence figures reach as high as 16% [2]. Tumour recurrence negatively affects patient prognosis with an estimated post-recurrence median survival of approximately one year [2,3].

In the pretransplant period, tumour features and recipient aspects (e.g., tumour vascular invasion, staging, and differentiation’s grade; viral aetiology of the liver disease, non-alcoholic fatty liver disease, time on the waiting list, and bridging therapies) are widely recognised as risk factors for HCC recurrence after LT [4]. Additionally, donor risk factors are also associated with tumour recurrence, including donor age, level of steatosis, and warm and cold ischaemia time [4,5]. Such risk combinations lead to a suboptimal organ utilisation with subsequent donor organ shortage. Another consequence is the allocation of donor livers with advanced risk profiles to medically fit recipients, frequently listed for HCCs or other cancers, including young patients with cholangiocarcinoma on the basis of a primary sclerosing cholangitis (PSC) [6,7]. Donor livers of marginal quality include, for example, advanced donor age or higher grades of steatosis, and are also summarized under the term extended criteria donors (ECD). Such organs transmit a higher risk of impaired function and posttransplant complications and also include pre-injured donor livers donated after circulatory death (DCD) with prolonged warm ischaemia times in some countries. The instinctive high-risk donor to low medical-risk recipient combination may place these patients at increased risk of higher tumour recurrence and, potentially, compromised HCC transplant outcomes [8].

Ischaemia-reperfusion injury (IRI) is considered a major determinant of the higher HCC recurrence rate associated with transplantation of ECD and/or ischaemic-damaged donor organs [9].

Machine perfusion (MP) of the liver has gained growing attention within the transplant community as a useful tool to alleviate IRI, to assess liver function prior to transplantation, and potentially recondition marginal organs [10,11]. Thus, MP may play an important role not only in increasing the utilisation of ECD organs, but also in improving the outcomes after transplantation of recipients with an HCC or other tumours. With this review article, we describe the underlying mechanisms of IRI and downstream inflammatory processes, which preform optimal conditions in the transplanted liver and recipient for tumour cells to resettle in the newly transplanted liver with subsequent cancer recurrence. We further highlight the potential protective effect of machine perfusion technology and discuss recent literature in this context.

## 2. Mechanisms of Ischaemia-Reperfusion Injury

Conventionally, after procurement, donor organs are cooled and stored in an ice box while being submerged in preservation solution as part of static cold storage (SCS). Despite the beneficial properties of these solutions, the lack of oxygen, which serves as the final electron acceptor during cellular respiration, inevitably interrupts the shuttling of electrons through the mitochondria electron transport chain (ETC) [11,12]. Several metabolites accumulate as a consequence, with succinate being the most prevalent [13]. The compromised ETC impairs oxidative phosphorylation and, finally, adenosine triphosphate (ATP) synthesis. Consequently, cellular ATP stores are depleted and, in order to provide cellular energy, anaerobic glycolysis is started and results in the production of lactate, tissue acidosis, and cell swelling. The failure of the Na^+^/K^+^-ATPase pump appears as a further consequence with depolarisation of the cell membrane and an influx of Ca2^+^/Na^+^ to the cytosol of the cells. The swelling and vasoconstriction of the sinusoidal endothelial cells (SEC) due to the cold temperatures and the release of vasoconstrictive substances (including endothelin and thromboxane-A2, not balanced by the vasodilatory nitric oxide (NO)), contribute to microcirculatory failure [12,14].

Paradoxically, during normothermic reperfusion, when the blood supply is restored after a period of hypoxia, the reoxygenation unveils the level of cellular damage. Under these conditions, the impaired ETC intensifies the leakage of electrons, and instigates entire reperfusion injury through an initial and immediate production of reactive oxygen species (ROS) at complex I [15,16,17]. This initial mechanism of injury appears at the same time in all cells undergoing reoxygenation at normothermic temperatures. Downstream to such mitochondrial injury, other cellular and subcellular structures contribute further to ongoing inflammation. For example, components of the injured cells release damage-associated molecular patterns (DAMPs), including DNA (high-mobility group box 1 (HMGB-1)) or heat shock proteins, which are recognised by cellular receptors (toll-like receptors (TLRs)) and trigger an activation of immune cells (e.g., Kupffer cells, dendritic cells) [18]. Resident Kupffer cells, in turn, release proinflammatory cytokines (e.g., tumour necrosis factor-alpha (TNF-α) and interleukin 1 (IL-1)) [14]. As a response to this initiation, SEC express adhesion molecules (such as E-selectin and the intercellular adhesion molecule 1 (ICAM-1)) and promote the recruitment of neutrophils, their adhesion, and migration in the extravascular space. The activated neutrophils perpetuate the production of ROS further and aggravate the inflammatory response with additional tissue damage. Various cells initiate their cell death programs (e.g., necrosis, apoptosis, or autophagy), in accordance with the level of overall injury [12,14]. SEC activation and sinusoidal neutrophil infiltration contributes to the microcirculatory dysfunction with repeat hypoxia [14,19].

The next important group of molecules involved in this general tissue inflammation is the Rho-associated protein kinase family (Rac1, ROCK, and Cdc-42), which regulates vascular constriction. Importantly, stellate cell constriction and neutrophil migration contribute further [20]. Inflammatory cell activation and infiltration are further promoted by other chemokines, including the C-X-C motif ligand 10 (CXCL-10) and matrix metalloproteinases (MMPs) [21]. CXCL-10 is a downstream product of the Damps-Toll-Like Receptor 4 (TLR-4) pathway of the innate immune system [22]. CXCL10 promotes proinflammatory gene induction [23], intra-graft recruitment of endothelial progenitor cells (EPCs) [24], and mobilisation of more regulatory T cells (Tregs).

The ischaemia and microcirculatory dysfunction associated with IRI cause the next period of relative tissue hypoxia and, consequently, expression of the hypoxia-inducible factor-1 (HIF-1). The HIF-1 has a subunit alfa (HIF-1α), which is oxygen-destructible and degrades under the normoxic conditions by the ubiquitin-proteasome system [25]. HIF-1α plays a protective role as a regulator of hypoxia-responsive genes, improving mitochondrial function and glycolytic pathways and mitigating ROS production and apoptosis [26]. Additionally, HIF-1α upregulates the transcription of molecules associated with angiogenesis, including vascular endothelial grow factor (VEGF), a well-known response to tissue hypoxia [25,26].

## 3. The Link between Ischaemia-Reperfusion Injury and Tumour Recurrence after Liver Transplantation

Various experimental models have shown that an alteration of the hepatic microenvironment caused by IRI promotes HCC recurrence after LT and leads to the mobilisation of progenitor cells and the development of more aggressive tumour phenotypes. Three main features were identified, which facilitate circulating tumour cells to resettle.

### 3.1. The Favourable Microenvironment

**Figure 1:**
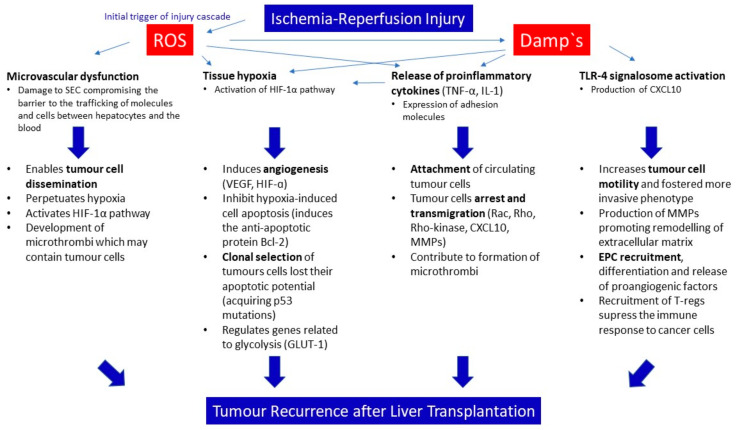
Underlying mechanism of reperfusion injury and the link to tumour recurrence. Ischemia-reperfusion injury was shown to be directly linked to tumour cell resettling and recurrence through creation of a favourable milieu for tumour cells to reconnect within tissues. Multiple intercellular pathways between donor and recipient tissue and the circulation are highlighted.

The IRI-associated SEC injury leads to a general microvascular dysfunction (endothelial cell swelling, unbalanced vasoconstriction, and neutrophil plugging) and compromises the barrier to the trafficking of molecules and cells between hepatocytes and the blood [27]. Severe impairment of endothelial structures plays a pivotal role during the acute phase of liver IRI and enables tumour cell dissemination in a later phase [21,27]. The disruption of microcirculation perpetuates tissue hypoxia and activates the HIF-1α pathway as a protective mechanism [25]. Hypoxia facilitates the survival of tumour cells and cancer growth. The upregulation of HIF-1α is another well-recognized promoter of tumour cell proliferation [28]. This is because HIF-1α regulates genes related to hypoxia-induced cell death (tumour suppressor gene *p53* and *Bcl-2* (B-cell lymphoma 2)), angiogenesis (VEGF), and glycolysis (glucose transporter-1 (GLUT-1)) [26]. Indeed, the lack of HIF-1α hampers the solid tumour growth and neo-vascularisation associated with an upregulation of VEGF stimulated by hypoxia [29]. VEGF itself induces angiogenesis and inhibits hypoxia-induced cell apoptosis by inducing the anti-apoptotic protein Bcl-2 [30]. Thus, HIF-1α is associated with a clonal selection of tumour cell variants, which have lost their apoptotic potential, favouring those with a more malignant phenotype (acquiring *p53* mutations) [31]. In addition, the microvascular dysfunction after IRI promotes the development of microthrombi, which may include tumour cells (Figure 1) [32].

### 3.2. A Higher Aggressiveness of Tumour Cells

More than 20 years ago, animal studies suggested the association between hepatic IRI and haematogenous liver metastases [33]. The attachment of circulating tumour cells to the vascular endothelium is discussed as the key step in this process. IRI-induced inflammatory cytokines, including TNF-α or IL-1, may promote the development of metastases in several types of cancer cells via the expression of adhesion molecules (e.g., e-selectin, ICAM-1, and vascular cell adhesion molecule 1 (VCAM-1)), which act as mediators for tumour growth [34,35,36]. Such molecules further promote the inflammatory microenvironment and facilitate tumour cell detention and transmigration into the extravascular space [37]. The inhibition of adhesion molecules through a specific neutrophil elastase inhibitor was shown to reduce the number of hepatic metastases in an experimental model of IRI and colorectal adenocarcinoma [37]. The expression of ICAM-1 on circulating tumour cells promotes their extravasation through an interaction of this adhesion molecule with β2-integrins on arrested neutrophils [38]. In addition, IRI also affects tumour cell invasion and migration through modulation of the Rho-family, regulators of cell motility, proliferation, and apoptosis [39]. The overexpression of Rac, Rho, and ROCK (Rho-kinase), found in rodent tumour tissues in the context of hepatic IRI, did correlate with infiltrative tumour growth and metastatic patterns [39]. The aggressive properties of these cancer cells were characterised by the overexpression of Rac1 in liver tumours and in intrahepatic and lung metastases [39]. In a rat hepatoma model, the use of a ROCK inhibitor suppressed cancer cell migration and reduced tumour recurrence after liver transplantation [40]. CXCL-10, a chemoattractant, which is upregulated during IRI, promotes macrophage infiltration in the liver and macrophage activation in tumours with more tumour invasion and infiltration to blood vessels after transplantation. In addition, CXCL-10 was shown to increase tumour cell motility and fostered a more invasive phenotype, with establishment of prolific stress fibres inside tumour cells (Figure 1) [41].

Another important class of enzymes to highlight here are matrix metalloproteinases (MMPs), which are involved in the remodelling of the extracellular matrix. In particular, an increased expression of MMP-9 was described during IRI [42,43]. MMP-9 enhances the recruitment and transmigration of neutrophils and T cells into the liver [43]. In a mouse model, IRI-induced expression of MMP-9 and predisposed the growth of micro-metastases of colorectal carcinoma in the liver, while the subsequent administration of MMP-inhibitors may reduce the metastatic tumour burden [44].

### 3.3. The Mobilisation of Progenitor Cells

The CXCL-10 pathway, previously described here, is also associated with EPC recruitment, differentiation, and neovascularisation. Pro-angiogenic factors elaborated by EPC may offer a favourable environment for post-LT tumour recurrence and metastases [24]. A clinical study with advanced unresectable HCC (a solid tumour with rich neo-vasculature) presented higher levels of circulating EPCs when compared with that of patients with less advanced disease. The authors conclude that, in patients with HCC, the levels of circulating EPCs may function as a prognostic marker [45]. Next, through an interaction with the surface receptor CXC-chemokine receptor-3 (CXCR-3), the molecule CXCL-10 was found to promote the recruitment of Tregs to the liver [22]. Whilst Tregs supress the immune response, support the induction of transplant tolerance, and prevent allograft rejection, they also may negatively affect the responses against tumours and promote tumour growth [46]. Thus, Tregs may help tumour cells to escape from the host immunosurveillance by their potent immunosuppressive activity [46]. Furthermore, in a mouse model of hepatic IRI, it was demonstrated that during liver graft injury the expression of TLR-4 and CXCL-10 is upregulated, with further recruitment of CXCR-3-positive Tregs into the liver. Such findings were paralleled by an inhibition of tumour recurrence after hepatic ischaemia-reperfusion (IR) injury achieved through a knockout of CXCL-10 and Treg-depletion (Figure 1) [22].

## 4. The Clinical Evidence for the Link between Advanced IR Injury and Tumour Recurrence after Liver Transplantation

To document the described impact of IRI on HCC recurrence after LT, a comprehensive literature review was done using various terms, including liver transplantation, hepatocellular carcinoma, recurrence, tumour recurrence, cancer recurrence, ischemia-reperfusion injury, and donor and recipient risk. The main focus was on clinical studies within the last 10 years, where authors provide a link between donor and recipient risk, IRI, and outcome including cancer recurrence and recurrence free survival (Table 1).

In a recent retrospective single-centre study with 195 patients transplanted for HCC, authors demonstrated that elevated liver enzymes (AST ≥1896 U/L) significantly increase the risk of HCC recurrence after LT in patients already within the Milan criteria (*p* = 0.035) [9]. Interestingly, the AST elevation was found to be more predictive than donor risk factors, despite the use of fairly good livers donated after brain death (DBD) (Table 1).

A large retrospective study, using the Scientific Registry of Transplant Recipients, analysed 9724 patients with HCC who received a LT [4]. In a multivariable Cox regression and competing risk analysis, donors older than 60 years (hazard ratio (HR) 1.38, 95% confidence interval (CI) = 1.10–1.73; *p* = 0.006), a donor history of diabetes (HR 1.43, 95% CI 1.11–1.83; *p* = 0.006), and donor body mass index ≥35 kg/m^2^ (HR 1.36, 95% CI 1.04–1.77; *p* = 0.023), were significantly associated with an increased rate of HCC recurrence. Expectedly, a prolonged donor warm ischaemia time (dWIT) was also described as a risk factor for an HCC recurrence (*p* = 0.025) (Table 1) [4].

Prolonged donor warm and cold ischaemia times (CIT) were linked with higher HCC recurrence rates post-LT [5,52,55]. Nagai et al. examined 391 patients with HCC [5]. In a multivariate Cox’s regression analysis, a CIT of >10 h and recipient WIT >50 min were identified as independent risk factors for overall HCC recurrence after LT (*p* = 0.03; HR = 1.9; *p* = 0.003; HR = 2.84, respectively). This finding was even more pronounced in liver recipients with aggressive HCC types, including a poor tumour differentiation, micro- and macrovascular invasion, and exceeding Milan criteria, or an alpha-fetoprotein (AFP) of >200 ng/mL [5]. Patients with CIT >10 h also showed significantly higher peak AST and ALT levels within the first week after LT (AST, *p* = 0.003; ALT, *p* = 0.01). The authors concluded that strategies to shorten ischaemia time may improve outcomes for those patients (Table 1) [5].

Such findings were paralleled by another study examining 103 LT patients with HCC [52]. Tumour relapse after LT was associated with prolonged mean CIT (468.0 vs. 375.5 min; *p* = 0.001) and recipient WIT (58.4 vs. 45.7 min; *p* = 0.001) [52]. Finally, this study also suggests that minimisation of ischaemia times may improve outcomes after LT in patients with HCC [52]. To test this hypothesis, the same group administered alprostadil, a prostaglandin E1 (PEG-1) analogue in the intensive care unit (ICU) after LT to mitigate the posttransplant IRI [55]. Markers of post-reperfusion injury were significantly lower in the PEG-1 group (Mean post-LT peak: AST, 581.7 vs. 780.7 IU/mL, *p* < 0.001; ALT, 559.6 vs. 701.4 IU/mL, *p* < 0.001). Importantly, the PEG-1 group had superior 3- and 5-year recurrence-free survivals (*p* = 0.003). The multivariate Cox regression analysis found the absence of PEG-1-treatment as the independent risk factor for early HCC recurrence within 12 months (HR = 5.3, 95% CI 1.06–26.5; *p* = 0.042) [55]. For patients exceeding the Milan criteria, alprostadil was an independent promoter of recurrence-free survival [55]. Overall, there are three retrospective studies based on the large United Network for Organ Sharing (UNOS) data set, with the most recent published in 2020, where DCD livers or donor warm ischemia time were shown to impact on HCC recurrence. Two single-centre studies, one from the USA and one from the UK, have, however, demonstrated that good DCD liver grafts with short donor warm ischemia times and an overall lower donor risk can achieve similar outcomes compared to standard DBD liver grafts [48,54,56]. The majority of clinical studies describe the HCC recurrence in general without a more detailed description of the exact site. Only one study, by Croome et al., showed that DCD liver recipients have a higher recurrence rate found first in the chest (43%) compared to DBD recipients with only 24%. In more than half of all DBD liver recipients with recurrence, the new HCC lesions were found in the transplanted liver [53].

Whilst robust evidence is lacking and some results remain controversial, different groups have demonstrated the link between donor livers that are more vulnerable to IRI, including DCD grafts and steatotic livers, or livers from living donors with the need to regenerate, and a higher tumour recurrence with subsequent inferior survival rates after LT [50,57,58].

## 5. The Potential Role of Machine Perfusion of the Liver on Tumour Recurrence

The increased utilisation of donor organs with a high risk of IRI and subsequent post-transplant complications (ECD organs) [59] has unveiled the limitations of traditional SCS, thereby renewing the interest in dynamic organ preservation techniques with the aim of reconditioning and assessing donor livers prior to LT [10,11]. Considering the mechanistical link between IRI and HCC recurrence and the clinical evidence, dynamic organ preservation approaches serve as promising strategies to reduce HCC recurrence. We describe in the next section the potential role of different perfusion techniques and their impact on IRI and HCC recurrence. Two main concepts are currently being explored in the clinical setting of liver transplantation. While hypothermic perfusion techniques aim to repair and improve livers prior to reperfusion under warm conditions at implantation, other techniques, including normothermic machine perfusion, replace cold ischemia and therefore reduce SCS time. The overall aim of all perfusion techniques is to evaluate liver function prior to transplantation [10,11].

### 5.1. Hypothermic Machine Perfusion of the Liver

The technology of hypothermic machine perfusion (HMP) has evolved throughout recent years and various concepts are currently being tested. To understand the potential impact of any perfusion technique, the underlying mechanisms of protection should be presented. Regarding HMP, several experimental and clinical studies are available in different organs [60,61,62]. It is widely known that any form of ischemia implies metabolic changes in mammalian tissues, which are, however, more significant during warm when compared to cold ischemia [13,63]. Due to the interruption of electron flow through the respiratory chain, succinate and other precursors accumulate, and metabolic substrates including ATP get lost [15,16,64,65,66]. Such features appear during ischemia and remain invisible until mammalian tissues undergo rewarming, where ROS are produced immediately at mitochondrial complex I, with a direct relation to a specific flavoprotein (flavin mononucleotide (FMNH_2_)) released from the same site in complex I [67,68,69,70,71,72]. The restoration of oxygen to ischemic tissues, at transplantation or during machine perfusion, will always induce ROS and FMN release from mitochondria, strongly depending on the organ quality and subsequent level of accumulated succinate molecules during ischemia [15,68,72]. ROS molecules in turn lead to mitochondrial membrane pores opening with ubiquitarian downstream injury and inflammation triggered by the release of various molecules, such as danger associated molecular patters (DAMPs, including mitochondrial DNA) and cytokines. Higher temperatures during reoxygenation induce higher amounts of released ROS and subsequent downstream molecules as shown by multiple studies with confirmation in kidneys, lungs, hearts, and livers at normothermic temperatures [73,74,75,76,77,78,79,80]. The overall aim in the setting of organ transplantation is therefore to prevent or reduce accumulated succinate in tissues prior to normothermic reperfusion [13].

The technology of HMP and hypothermic oxygenated perfusion (HOPE) of kidneys and livers was developed by different groups over the past 50 years, who repeatedly demonstrated that mitochondria recharge ATP more effectively at hypothermic temperatures and that cold oxygenation triggers succinate metabolization [73,75,81]. Importantly, during cold reoxygenation, such metabolic events are not paralleled by significant ROS release, which does usually occur during reoxygenation under normothermic conditions. In addition, mitochondria release very limited FMN [67]. The protection of mitochondria appears, therefore, as key feature of HMP to prevent reperfusion injury and subsequent inflammation with organ dys- or nonfunction [75,76,81,82]. HMP was not only shown to protect from hepatocyte injury with lower enzyme release and better function, a protection from biliary complications found in HMP-treated liver grafts was shown by different groups [76,81,83,84]. In addition, eight retrospective clinical studies have demonstrated a reduction of biliary complications by HMP. Such findings were recently summarised and paralleled by a meta-analysis, with a significantly lower overall incidence of biliary complications after LT in livers treated with previous hypothermic perfusion. Five randomized controlled trials are currently being completed and their results will include biliary complications as primary or secondary endpoints [61].

In addition to normothermic perfusion techniques, organ viability can be assessed during cold oxygenation, as recently demonstrated [67]. Here, mitochondrial markers of injury and function are used and include the FMN, released from complex I and NADH, a marker of complex I function. In 2013, a Zurich group demonstrated a switch in mitochondrial function within the first 60 min of HOPE treatment, with a subsequent metabolism of NADH as a marker of mitochondria repair and function [85]. Online fluorometry of HOPE perfusate enables the assessment of mitochondrial metabolism “life” during perfusion, allowing for the prediction of liver function before decision making and graft implantation [67,68,69,72]. Importantly, FMN correlated with graft loss, hospital stay, and overall complications [76,86]. Such online assessment of the mitochondrial metabolism is currently validated in other large liver cohorts and other organs, including kidney, heart, and lungs [67,87,88].

The role of mitochondria-derived oxidative injury has gained increasing interest in various processes including aging and cancer growth [89,90]. As described above, a number of biological mechanisms of inflammation have been described to link cancer recurrence with initial IR injury in the setting of liver transplantation [21,91]. Virchow identified the association between tissue inflammation and tumour development in 1863, showing tissue inflammation as the underlying cause [92]. Since those early times, a multifactorial network of signals, primarily designed to “repair”, has been identified as a response to an initial mitochondria-induced ROS release at reperfusion. Such response appears paradoxical because inflammation appears as the main promoter of cancer development and recurrence [21,51,91,93,94]. In this context the overall goal should be the early inhibition of an inflammatory reaction in the setting of any form of solid organ transplantation.

The HOPE technique is currently used in extended grafts, including DCD and steatotic livers, or in combinations of other risk factors, such as advanced donor age and cold ischemia time [76,84,95,96]. The dominant target here is to limit the amount of danger signals released by mitochondria upfront, including ROS and mitochondrial DNA, to reduce the inflammasome activation [67,76,97]. For example, He et al. demonstrated that HOPE treatment protects DCD livers through an inhibition of the TXNIP/NLRP-3 inflammasome, an oxidative stress dependent signalling pathway found during reperfusion [98,99]. Recent data from a Zurich group showed that HOPE treatment protects liver recipients from HCC recurrence, despite the use of extended human DCD liver grafts. Importantly, recipients of a DBD liver graft, with a presumed lower donor risk, experienced HCC recurrences 4-times more frequently compared to higher risk DCD livers that underwent additional HOPE treatment. Of note, recipients of HOPE-treated livers were found with a lower systemic inflammation when compared to DBD liver recipients. Such findings parallel earlier reports in models of liver and kidney transplantation, where a significant protection from innate immune response and acute graft rejection was conveyed by HOPE [47,78,100,101]. Based on the anti-tumour effect of HOPE, a routine application of cold oxygenated perfusion in all livers transplanted into HCC recipients would be suggested, independent of the donor type [102].

### 5.2. Normothermic Machine Perfusion of the Liver (NMP)

Nomrothermic perfusion techniques represent a different concept and imitate the physiological conditions, thereby recovering the metabolism of the donor organ and offering nutrients and oxygen at 37 °C. When applied instead of cold storage, NMP abbreviates the hypoxic period, preventing further ATP depletion [11]. Additionally, various parameters of liver injury are currently assessed during NMP to provide viability testing before liver implantation [103,104,105]. Although the ultimate goal of NMP technology is to offer therapeutic interventions for organ recondition during preservation, solid organs have to overcome the initial IRI on the perfusion device first, before the effect of a specific treatment agents can be seen [80]. Several new machines are currently under exploration to achieve a prolonged perfusion time of several days, which is needed to overcome IRI and to treat specific conditions in the livers. Very sophisticated devices are required, which include dialysis and, for example, diaphragm replacement [106].

A recent randomized controlled trial has assessed the impact of NMP in DBD and low risk DCD donor livers [107]. Nasralla et al. demonstrated in 121 NMP-perfused livers a 50% lower plasma AST in the recipient within the first seven postoperative days, compared to those with SCS grafts [107].

Two different approaches have been used to apply NMP in clinics. First, an immediate NMP after liver procurement with subsequent device transport and second, an endischemic NMP in the recipient centre with acceptance of a few hours of cold ischemia before starting the normothermic perfusion [11,107]. There is recent evidence that this endischemic approach may yield good outcomes in low risk DCD donor livers [108], while the recent work from Cambridge demonstrated inferiority of the endischemic NMP approach in DCD livers with advanced risk [107,109,110]. The feasibility of complete elimination of hypoxia via continuous NMP to prevent postreperfusion syndrome and IRI via ischaemia-free organ transplantation (IFOT), although it appears to be clinically possible, is challenging and limited. The IFOT approach will only be applicable in DBD organs [111]. Whilst in routine clinical practice minimisation of CIT is desirable, it is not always feasible because of logistics.

Although the replacement of cold ischemia time by NMP was shown to protect the organ from further ATP depletion and limits the accumulation of metabolic waste products [112], experimental models have also demonstrated oxidative tissue injury and activation of the inflammatory immune response during NMP [75]. Importantly, such IRI with downstream inflammation starts immediately when human tissues become rewarmed or perfused under normothermic conditions, either in situ at implantation or on a perfusion device, irrespective if leukocytes and platelets are present or not in the perfusion circuit [75]. An induction of inflammatory pathways was shown in different organs. Zhang et al. compared NMP with SCS in a reduced-size LT pig model and confirmed the presence of serum markers of oxidative tissue damage (malondialdehyde) and inflammatory cytokines (TNF-α, IL-1, IL-6) postoperatively [113]. In order to provide the complete picture, it would be of interest not only to measure the plasma levels of inflammatory markers, which were significantly lower in the NMP group, but also to quantify the same markers in the perfusates obtained from the device [113]. In addition, NMP promoted a significant reduction of molecules associated with apoptosis (cytochrome C, caspase 3) and cytokines and chemokines synthesis and secretion (NF-κB; p65) [113].

Therapeutic interventions during NMP, such as the administration of cytoprotective and/or metabolic-modulating agents, may be used to treat IRI, although further studies are still needed to conclusively prove this theory. A recent study, investigating the effect of the pharmacological modulation of the lipid metabolism of discarded donor human livers during NMP, demonstrated the feasibility of the downregulation of markers for oxidative tissue injury (4-hydroxynonenal), a reduction in the activation of immune cells (CD14; CD11b), and the release of inflammatory cytokines in the perfusate (TNF-α, IL-1β) [114]. An experimental rat model suggested recently the feasibility of using NMP as a delivery method of cy3-labeled *p53* small interfering (si)RNA to silence the *p53* tumour suppressor gene in order to prevent apoptosis and mitigate IRI [115].

Goldaracena et al. compared SCS with NMP and an anti-inflammatory cocktail (alprostadil, n-acetylcysteine, carbon monoxide, sevoflurane, and sub-normothermic temperature (33 °C)) in a pig transplant model [116]. The authors observed a reduction in AST levels and inflammatory cytokines (IL-6, TNF-α, galactosidase) during perfusion in the anti-inflammatory group, which was, however, not sustained after transplantation. Of note, the recipient mortality was comparable between the experimental groups [116].

Future therapeutic interventions during NMP, tackling specifically the mechanistical links connecting IRI and tumour recurrence after LT, is certainly an interesting area of research. Supplementation of NO precursors, such as L-arginine or chemical destabilisation of HIF-1α using the heat shock protein 90 inhibitor 17-DMAG may improve microvascular function [27]. In addition, experimental animal models have already indicated a reduced tumour recurrence could be achieved through pharmacological modulation of IRI and properties of tumour cells (such as migration and invasion) with the administration of Rho-associated kinase and MMP-9 inhibitors [40,44]. Finally, pharmacological interferences within the CXCL-10/CXCR-3 pathway might reduce liver tumour recurrence and metastases within the scenario of IRI via a fall in circulating EPCs, as suggested also by an experimental animal model using FTY720 treatment [117].

Despite such promising markers, the most effective strategy to prevent tumour recurrence could be a significant reduction of reperfusion injury prior to organ implantation. Based on the current literature, various hypothermic oxygenated perfusion techniques offer this to the recipients [61,118].

A combination of sequential HOPE and NMP may offer the further benefit of additional viability assessment during NMP [119]. This method could be especially advantageous if mitochondrial markers (FMN or NADH) appear too high during HOPE in high-risk organs. A recent preclinical study identified a lower expression of markers of oxidative tissue injury (4-hydroxynonenal, cluster of differentiation (CD)-14) and less inflammation (CD11b, VCAM-1) in livers submitted to a combined HOPE and NMP protocol, compared with livers that underwent NMP alone [119]. Whilst this approach seems promising, with similar findings also reported by another preclinical study [81] and a clinical trial [120], robust evidence via a randomised clinical trial is missing.

Oldani et al. assessed the impact of HOPE or NMP in a rodent model of DCD liver transplantation with 1 h of WIT and subsequent HCC cell injection after reperfusion [121]. Compared to transplanted fresh livers, DCD-LT developed a higher total tumour volume, confirming the impact of IRI on tumour cell growth. However, both perfusion approaches, HOPE and NMP, did not reduce the tumour occurrence, total tumour volume, or overall survival in this specific model [121]. However, the DCD model used here did not represent the clinical situation very well and results of this study and their interpretation need further confirmation in other experimental and clinical studies [121].

## 6. Summary

Current evidence suggests that machine liver perfusion offers superior organ preservation over SCS, which is likely to be associated with the alleviation of the detrimental effects of IRI [10,11].

Based on the literature, donor factors and severe IRI negatively affect HCC recurrence following LT [21,23,24]. This topic is of great interest to the transplant community because of the frequent allocation of ECD organs to HCC patients on the waiting list, who are usually less sick but then experience compromised outcomes. Thus, machine perfusion of the liver may offer a not well-explored benefit for the reduction of post-LT HCC recurrence rates. Clinical evidence has already started to highlight that hypothermic oxygenated perfusion (HOPE) may protect recipients not only from IRI and posttransplant complications but also from cancer recurrence, which appears inevitably linked to organ quality. Importantly, most of the experimental data available so far deserves careful consideration, and further clinical studies are required.

## Figures and Tables

**Table 1:** Clinical studies linking ischaemia-reperfusion injury and recurrence of hepatocellular carcinoma after liver transplantation.

First Author/Year Publication/Reference	Database/Centre	Donor Type	Number of Subjects	Preservation Method	Donor and Recipient Risk Factors Associated with Outcomes	Parameters Associated with IRI	Main Findings/Conclusion
**Clinical studies with Machine Perfusion**							
Mueller M, et al., 2020, [47]	Two-centre comparison (UK, Switzerland)	DBD vs. DCD with HOPE	70; 70	Hypothermic oxygenated perfusion (HOPE)	Transplantation with non-perfused grafts (DBD), advanced HCC risk (outside Milan, UCSF, and Metroticket II)	ALT release, INR, CRP	4-fold higher tumour recurrence rate in un-perfused DBD livers compared to HOPE-treated DCD cohort (25.7% vs. 5.7%, *p* = 0.002). 5-year tumour-free survival of 92% HOPE-treated DCD livers, despite high risk DCD donor livers and advanced HCC risk. DCD grafts can be used for recipients with higher risk HCC tumours (outside Milan, outside UCSF, and Metro-ticket II) if treated with hypothermic oxygenated perfusion (HOPE) prior to transplantation
**Clinical studies with Standard Cold Storage**							
Silverstein J et al., 2020, [48]	UNOS	DBD vs. DCD	6996; 567	SCS	DCD organs (low cumulative risk, short cold ischemia time of 6 and 5.4 h; young median donor age with 45 and 33 years), donor age, DRI, MELD	–	Liver transplantation with DCD liver grafts was an independent predictor of mortality. Differences in survival were observed in subgroups with higher risk of recurrence, including RETREAT score >4, AFP > 100 ng/mL, and viable tumours on last imaging. Donor or graft quality and HCC parameters impact on outcomes
Martinez-Insfran A.L. et al., 2019, [49]	Single centre (Europe)	DBD vs. DCD	18; 18	SCS	Cold ischemia time (overall low risk DCD grafts, short donor warm and cold ischemia times)	AST release, ALT release, prothrombin time	DCD liver recipients have inferior patient survival, not significant with *p* = 0.266 and *n* = 18 in both groups; low risk DCD grafts can be used for standard HCC recipient
Grąt M, et al., 2018, [9]	Single centre (Europe)	DBD	195	SCS	Cold ischemia times, recipient WIT (implantation time)	AST release, LDH release, GGT, Peak Bilirubin, INR	AST ≥1896 U/L increases the risk of HCC recurrence after LT, already in patients within Milan criteria (*p* = 0.035)
Khorsandi SE et al., 2016, [50]	Single centre (UK)	DBD vs. DCD	256; 91	SCS	DCD vs. DBD grafts, donor warm and cold ischemia times, HCC risk factors	AST release, INR	DCD livers release more transaminases (AST) and have an impaired function (INR), recipients of good quality DCD livers have a similar risk of HCC recurrence compared to standard DBD donor liver recipients
Orci LA, et al., 2015, [51]	UNOS	DBD vs. DCD	9724	SCS	Donor WIT in DCD organs, donor age, donor BMI	–	Donor age >60 years and donor WIT was a risk factor for an increased HCC recurrence (*p* = 0.025)
Nagai et al., 2015, [5]	Two-centre analysis (USA)	DBD	391	SCS	Cold ischemia time and recipient WIT (implantation time), outside Milan, micro/macrovascular invasion, AFP >200 ng/mL; poor differentiation	AST release, ALT release	CIT >10 h and recipient WIT >50 min were independent risk factors for HCC recurrence after LT (*p* = 0.03; HR = 1.9; *p* = 0.003; HR = 2.84, respectively)
Kornberg A, et al., 2015, [52]	Single centre (Europe)	DBD	106	SCS	Cold ischemia time and recipient WIT, HCC risk factors	AST release, ALT release	Prolonged mean CIT (468.0 vs. 375.5 min; *p* = 0.001) and recipient WIT (58.4 vs. 45.7 min; *p* = 0.001) were associated with HCC recurrence after LT, protective effect of prostaglandin on recurrence free survival and HCC recurrence was more pronounced in recipients outside Milan criteria (*p* < 0.001)
Croome et al., 2015, [53]	Single centre (USA)	DBD vs. DCD	340; 57	SCS	Cold ischemia time and donor WIT, recipient AFP, recipient disease severity	–	Good DCD livers have a similar risk of HCC recurrence compared to standard DBD donor livers
Croome K et al., 2013, [54]	UNOS	DBD vs. DCD	5638; 242	SCS	Warm ischemia time, cold ischemia time, recipient age, lab MELD	–	DCD liver recipients have a higher risk of HCC recurrence compared to DBD graft recipients, recipients of livers with a warm ischemia time of >15 min, or a cold ischemia time of > 6 h 20 min had lower survival rates

## Abbreviations

AMP	Adenosine monophosphate
ADP	Adenosine diphosphate
ALT	Alanine aminotransferase
AS	Anastomotic strictures
AST	Aspartate-aminotransferase
ATP	Adenosine triphosphate
CD-68	Cluster of differentiation 68
CIT	Cold ischemia time
CS	Cold storage
XCL-10	C-X-C motif ligand 10
CXCR-3	CXC-chemokine receptor-3
DAMPs	Danger associated molecular patterns
DBD	Donation after brain death
DBQ	Decylubiquinone
DCD	Donation after circulatory death
D-HOPE	Dual hypothermic oxygenated perfusion
dWIT	Donor warm ischemia time
EAD	Early allograft dysfunction
ECD	Extended criteria donor
ECMO	Extracorporeal membrane oxygenation
FAD	Flavin adenine dinucleotide
FMN	Flavin mononucleotide
GSH	Glutathione
HA	Hepatic artery
HAR	Hexaammineruthenium
HAT	Hepatic artery thrombosis
HCC	Hepatocellular carcinoma
HIF-1α	Hypoxia-inducible factor-1α
HMGB-1	High mobility group box-1 protein
HMP	Hypothermic machine perfusion
HOPE	Hypothermic oxygenated perfusion
H&E	Hematoxylin and Eosin
HR	Hazard ratio
IC	Ischemic cholangiopathy
ICAM-1	Intercellular adhesion molecule-1;
ICU	Intensive care unit
IFOT	Ischaemia-free organ transplantation
IMP	Inosine monophosphate
IFOT	Ischaemia-free organ transplantation
KC‘s	Kupffer cells
LDH	Lactate dehydrogenase
LT	Liver transplantation
MELD	Model of end stage liver disease
MMP	Matrix metalloproteinases
MPS	Machine perfusion solution
MPT pore	Mitochondria permeability transition pore
NAD/NADH	Nicotine adenine dinucleotide (oxidized/reduced)
NADPH	Nicotinamide adenine dinucleotide phosphate hydrogen
NAS	Non-anastomotic strictures
NRP	Normothermic regional perfusion
NMP	Normothermic machine perfusion
OAA	Oxaloacetate
OLT	Orthotopic liver transplantation
PEG-1	Prostaglandin E1
PMH	Past medical history
PNF	Primary non function
PV	Portal vein
RET	Reverse electron flow
ROS	Reactive oxygen species
SDH	Succinate dehydrogenase
SEC	Sinusoidal endothelial cells
TLR-4	Toll-like-receptor-4
Tregs	Regulatory T cells
VCAM-1	Vascular cell adhesion molecule 1
VEGF	Vascular endothelial grow factor
8-OHdG	8-hydroxy-2-deoxy Guanosine
